# Interaction between personality traits and cerebrospinal fluid biomarkers of Alzheimer’s disease pathology modulates cognitive performance

**DOI:** 10.1186/s13195-017-0235-0

**Published:** 2017-02-02

**Authors:** Domilė Tautvydaitė, Deepti Kukreja, Jean-Philippe Antonietti, Hugues Henry, Armin von Gunten, Julius Popp

**Affiliations:** 1 Department of Psychiatry, Service of Old Age Psychiatry, Lausanne University Hospital (CHUV), Ch. de Mont-Paisible 16, CH-1011 Lausanne, Switzerland; 2 Institute of Psychology, University of Lausanne, Lausanne, Switzerland; 3 Service of Biomedicine, Lausanne University Hospital (CHUV), Lausanne, Switzerland

**Keywords:** Premorbid personality, Cerebrospinal fluid biomarkers, Alzheimer’s disease, Mild cognitive impairment, Cognition

## Abstract

**Background:**

During adulthood, personality characteristics may contribute to the individual capacity to compensate the impact of developing cerebral Alzheimer’s disease (AD) pathology on cognitive impairment in later life. In this study we aimed to investigate whether and how premorbid personality traits interact with cerebrospinal fluid (CSF) markers of AD pathology to predict cognitive performance in subjects with mild cognitive impairment or mild AD dementia and in participants with normal cognition.

**Methods:**

One hundred and ten subjects, of whom 66 were patients with mild cognitive impairment or mild AD dementia and 44 were healthy controls, had a comprehensive medical and neuropsychological examination as well as lumbar puncture to measure CSF biomarkers of AD pathology (amyloid beta_1–42_, phosphorylated tau and total-tau). Participants’ proxies completed the Revised NEO Personality Inventory, Form R to retrospectively assess subjects’ premorbid personality.

**Results:**

In hierarchical multivariate regression analyses, including age, gender, education, APOEε4 status and cognitive level, premorbid neuroticism, conscientiousness and agreeableness modulated the effect of CSF biomarkers on cognitive performance. Low premorbid openness independently predicted lower levels of cognitive functioning after controlling for biomarker concentrations.

**Conclusion:**

Our findings suggest that specific premorbid personality traits are associated with cerebral AD pathology and modulate its impact on cognitive performance. Considering personality characteristics may help to appraise a person’s cognitive reserve and the risk of cognitive decline in later life.

## Background

With the rising number of older people suffering from Alzheimer’s disease (AD), it is of high clinical importance to investigate its early clinical stages (i.e. mild cognitive impairment (MCI)) as well as to identify lifestyle-related factors that influence the relationship between cerebral pathology and cognitive impairment. Patients with MCI are characterized by a noticeable decline in cognitive abilities, which is relatively greater than normal age-related change observed in the healthy population, and have an increased risk of developing AD dementia during the following year. The underlying pathologies are heterogeneous and only some patients have cerebral AD pathology as the main cause of cognitive impairment. Cerebrospinal fluid (CSF) AD biomarkers reflect the cerebral accumulation of specific pathology; that is, the extracellular plaques containing amyloid beta (Aβ) [1, 2] and the neurofibrillary tangles, associated with tau protein abnormalities and neurodegeneration [3, 4]. Compared with controls, mildly demented AD patients show decreased Aβ_1–42_ levels and elevated tau protein levels in CSF [5, 6]. Studies show that these neuropathological changes precede the symptoms of clinical dementia by years [7, 8], and thus tau, phosphorylated tau (*p*tau) and Aβ_1–42_ in CSF can be considered a valuable detector of AD at a very early stage.

The risk of dementia has been reported to be associated with specific premorbid personality characteristics [9]. Personality is described as a more or less stable organization of character, temperament and intelligence of a person that determines adaptation to the environment [10]. Personality traits represent individual tendencies to think, feel and behave in certain ways that affect our interactions with the external world [11]. Because personality influences lifestyle patterns such as health habits, cognitive activity and social relationships [12, 13], and these factors in turn are related to the risk of developing dementia in later life [14–16], personality characteristics may represent important determinants of dementia risk.

In Alzheimer’s type of dementia, the age of onset and the magnitude of cognitive impairment may depend on the individual’s capacity to compensate cerebral pathology and dysfunction, referred to as cognitive reserve [16]. Beginning as early as in young adulthood, personality characteristics may contribute to cognitive reserve by their influences on lifestyle and health-related behaviour. A resilient personality profile may be associated with lower risk of clinical dementia in persons with AD neuropathology. Wilson et al. [17] have shown an association between higher levels of conscientiousness and reduction in MCI and AD incidence and reduced cognitive decline. Likewise, two comprehensive meta-analyses showed consistent evidence for associations between higher premorbid neuroticism and increased risk of dementia, while higher levels of conscientiousness were demonstrated to act as protective factors for dementia incidence [18, 19]. These studies have not included evaluation of cerebral AD pathology, however. One prospective autopsy study showed that in subjects confirmed to have AD neuropathology, greater scores on conscientiousness and lower scores on neuroticism were associated with reduced risk or delay to develop clinical dementia [20].

Information regarding personality traits is accessible through questionnaires completed by patients’ relatives, and does not involve any invasive techniques. Evaluating subjects’ personality may add significant information on the risk of AD dementia and favour the prevention of cognitive decline. The possible influence of premorbid personality characteristics on the relationship between cognitive functioning and AD pathology, as measured by CSF biomarkers at early clinical disease stages, has not yet been questioned. With this study we aim:

1.  to explore the relationship between premorbid personality traits and cognitive functioning in cognitively impaired patients and healthy older subjects; and
2.  to investigate whether specific personality traits modulate the relationship between cognitive performance and cerebral AD pathology as measured by CSF biomarkers in subjects with MCI or mild dementia and in subjects with normal cognition.

## Method

### Subjects

One hundred and ten community-dwelling participants were included in this study, of whom 44 were cognitively healthy volunteers and 66 had cognitive impairment, either MCI (*n* = 57) or mild dementia AD (*n* = 9) (see Table 1 for a detailed sample description). The participants with cognitive impairment were recruited among patients attending the Memory Clinics of the Department of Psychiatry and the Department of Clinical Neurosciences at Lausanne University Hospital, and met the diagnostic criteria for MCI [21] or mild dementia [22]. The patients had no major psychiatric or neurological disorders, nor substance abuse or severe or unstable physical illness that explained cognitive impairment. Control subjects had no history, symptoms or signs of relevant psychiatric or neurologic disease and no cognitive impairment. Healthy subjects were recruited in the community through journal announcements and word of mouth. All participants had a comprehensive medical, psychiatric, neuropsychological and psychosocial evaluation, as well as brain MRI or CT scans and venous and lumbar puncture. The MRI and CT scans were used in order to exclude patients with cerebral pathologies possibly interfering with cognitive performance, including relevant vascular damage.

**Table 1:** Demographics and descriptive statistics

		Control	Cognitive impairment	Statistical test	*P*
		(*n* = 44)	(*n* = 66)		
Gender	Males	14 (31.8%)	30 (45.5%)	Chi-square	0.155
	Females	30 (68.2%)	36 (54.5%)		
Age	Mean	66	74	*t*	<0.001
	SD	6.57	6.54		
Education level	≤9 years	3 (6.8%)	11 (16.7%)	Chi-square	0.113
	10–12 years	22 (50.0%)	34 (51.5%)		
	>12 years	19 (43.2%)	21 (31.8%)		
CDR SoB	Mean	0.23	1.96	*t*	<0.001
	SD	0.1	2.16		
HAD Depression score	Mean	3.79	4.18	*t*	0.55
	SD	3.56	3.06		
HAD Anxiety score	Mean	6.79	6.45	*t*	0.65
	SD	4.24	3.2		
QPC score	Mean	1.56	2.54	*t*	<0.01
	SD	1.5	1.89		
IQCODE score	Mean	3.067	3.467	*t*	<0.001
	SD	0.42	0.54		
APOEε4 carriers	No	36 (81.8%)	36 (54.5%)	Chi-square	<0.01
	Yes	8 (18.2%)	29 (43.9%)		
Aβ_1–42_ pg/ml	Median	1053	668.75	*U*	<0.001
	IQR	289.6	357.8		
*tau* pg/ml	Median	209.3	394.35	*U*	<0.001
	IQR	115.4	361.3		
*p*tau-181 pg/ml	Median	46.5	62.3	*U*	<0.001
	IQR	25.2	44.5		
*tau*/Aβ_1–42_ pg/ml	Median	0.219	0.554	*U*	<0.001
	IQR	0.115	0.765		
ptau-181/Aβ_1–42_ pg/ml	Median	0.047	0.101	*U*	<0.001
	IQR	0.02	0.097		

*CDR SoB* Clinical Dementia Rating Sum of Boxes score, *HAD* Hospital Anxiety and Depression Scale, *APOEε4* epsilon 4 allele of Apolipoprotein E, *QPC* cognitive complaints questionnaire, *IQCODE* Informant Questionnaire on Cognitive Decline in the Elderly, *Aβ* amyloid beta, p*tau* phosphorylated tau, SD standard deviation, *IQR* interquartile range, *U*, Mann–Whitney *U* statistic

### Procedure

The study was approved by the Ethics Committee of the canton of Vaud, Switzerland. The aims of the research project were clearly explained to all participants and the informed written consent to participate in the study was administered and signed by all.

Diagnosis of MCI or mild AD dementia was based on clinical and neuropsychological evaluation as well as on psychosocial and functional assessment (see later) and was made at a consensus conference of psychiatrists, neuropsychologists and/or neurologists prior to inclusion into the study. MCI was diagnosed according to widely used consensus recommendations [21]. Participants in this group had memory impairment (<–1.5 standard deviation (SD) below the means adjusted for gender, age and education in the verbal memory task of the Buschke Double Memory Test [23]) and/or impairment in another cognitive domain, and a Clinical Dementia Rating (CDR) [24] score of 0.5. The diagnosis of probable AD dementia was based on the clinical diagnostic criteria for probable dementia due to AD according to recommendations from the National Institute on Aging and Alzheimer’s Association [25] and DSM-IV criteria for dementia of Alzheimer type [22]. Participants in this group had a CDR score of 1. Participants without cognitive impairment had no history or evidence of cognitive decline, and their CDR score was 0.

The CDR is a semi-structured, clinician-rated interview with the patient and an appropriate informant, widely used to assess the progression of dementia. It is based on the ratings of the patient’s cognitive and functional impairment in six domains usually affected in AD: memory, orientation, judgement and problem-solving, community affairs, home and hobbies, and personal care [24]. Scores in each of area range from 0 to 3, representing “none” to “severe” impairment. CDR has a very high inter-rater reliability [26] and thus appears to be a reliable and valid measurement for assessing cognitive performance stages in dementia.

### CSF biomarkers and APOE genotyping

Venous and lumbar punctures were performed between 8:30 and 9:30 am after overnight fasting at the recruiting memory centres. CSF was collected by lumbar puncture using a standardized technique with a 22 G “atraumatical” spinal needle while the patient was sitting or lying [27]. Samples of 10–12 ml of CSF was obtained for analysis using polypropylene tubes. Routine CSF cell counts and protein quantification were performed. The remaining CSF was centrifuged, frozen in aliquots and stored at –80 °C until assayed. We measured Aβ_1–42_, *tau* and *p*tau-181 concentrations with ELISA kits, using commercially available assays (Innogenetics/Fujirebio, Gent, Belgium). Carrying the APOEε4 allele may influence the relationships between personality, AD pathology, age and clinical manifestations [28] at very early disease stages [29, 30]. Accordingly, the APOE genotype was determined (using the LightCycler; Roche Diagnostics, Basel, Switzerland) and considered in all main analytical steps. Analyses were performed by operators who were blind to all clinical information.

### Neuropsychological and functional assessment

Neuropsychological tests were used to assess cognitive performance in the domains of memory (spontaneous and cued-recall 48-items task [31]), executive function—a verbal fluency task (categorical and literal fluency in 2 min), a flexibility task (the Trail Making Test A and B [32]) and an inhibition task (the Stroop test [33])—and visuospatial functions (CERAD copy image test). The MMSE [34] was used to assess participants’ cognitive level. Depression and anxiety were assessed using the Hospital Anxiety and Depression (HAD) scale [35]. A cognitive complaints questionnaire (Questionnaire de Plainte Cognitive (QPC) [36, 37]) was employed to evaluate participants’ memory failures and cognitive complaints.

Psychosocial and functional assessment included the ADL [38] and instrumental ADL [39], the NPIQ [40] and the IQCODE [41] questionnaires, completed by family members of the participants. All tests and scales are validated and widely used in the field.

### Personality assessment

Patients’ proxies were asked to complete the Revised NEO Personality Inventory, Form R (NEO-PI-R [42]), which is based on the Five-Factor Model (FFM) [43]. This dimensional personality model, derived from factor analyses performed on a large number of self-reports and peer reports on personality-relevant adjectives and questionnaire items [43], revealed five dimensions describing personality: neuroticism, extraversion, openness to experience, agreeableness and conscientiousness. Each of these dimensions is composed of six subcomponents, or facets (see Table 3 in Appendix for description of the domain and facet scales). It was suggested that normal and pathological personality may be differentiated by the extremeness of scores on the dimensions of the FFM [43]. The NEO-PI-R is a questionnaire composed of 240 items and used for peer ratings which has resulted from comprehensive research on personality change and stability, and has well-established reliability and validity data in older populations [44], and was validated with a French-speaking sample [45]. For this study we used the informants’ ratings, because subjects with cognitive impairment may provide less reliable information about their personality due to memory difficulties and poor insight and judgement [46]. Premorbid personality was assessed with the NEO-PI-R, Form R where the subjects’ proxies were asked to describe the participants’ personality as it was remembered to be 5 years prior to the beginning of the first symptoms.

### Statistical analyses

Data were analysed using SPSS version 20 and R [47]. To characterize and compare the cognitively impaired (MCI and mild AD dementia considered together, CI) group and the control group, we applied descriptive statistics [48], the Mann–Whitney test [49] for non-normally distributed data, and Student’s *t* tests for independent samples. Comparison of premorbid personality traits between clinical and control subjects was performed using Welch’s *t* test [50]. To screen for relations between premorbid personality traits and cerebral pathology we counted correlations for each NEO-PI-R, Form R domain and each biomarker. Further, four-stage multiple hierarchical regressions were conducted for the CI and control groups combined to determine whether any of the NEO personality traits contributed significantly to cognitive performance, represented by the CDR SoB score. All observations that were shown by Cook’s distance [51] to be influential were eliminated from this analysis. We first included age, gender, education level, APOEε4 expression and cognitive status (Controls/CI) in our hierarchical regression models to control for those variables that may have an impact on the cognitive performance. In the second step we examined the concentration of CSF biomarkers. Then, each of the five main NEO-PI-R personality domains was added separately in order to assess the influence of personality traits on predicting cognitive performance. Finally, we considered interactions between each dimension of premorbid personality and concentration of CSF biomarkers. Different regression models were compared and domain indices were calculated.

## Results

The descriptive statistics of the sample are presented in Table 1. The CI and control groups differed significantly in age, APOE ε4 status and CDR SoB score. There were significantly more females than males in the control group, although gender distribution in the clinical group was equal. HAD depression and anxiety scores were similar in both groups.

### Simple group comparisons

Welch’s *t* test performed on the informant-report data for each of the five personality dimensions yielded a significant difference in openness to experience scores in both the control (M = 107; SD = 17.27) and CI (M = 94; SD = 17.22) groups (*t*(92.12) = 15.318, *p* < 0.001). Premorbid personality traits in domains of neuroticism, extraversion, agreeableness and conscientiousness did not differ between the two experimental groups (*t*(87.41) = 0.96, *p* = 0.33; *t*(107.61) = 1.58, *p* = 0.21; *t*(101.16) = 0.09, *p* = 0.76; and *t*(90.06) = 1.19, *p* = 0.28 respectively). Moreover, among the personality domains, only openness to experience was significantly correlated with MMSE score (*r* = –0.243, *p* < 0.05) and CDR SoB score (*r* = 0.325, *p* < 0.01) in the group with cognitive impairment.

### Personality characteristics and CSF biomarkers

We applied Kendall’s tau [52] correlation analysis for nonparametric data to investigate associations between premorbid personality traits and concentrations of CSF biomarkers (see Table 2). Because the biomarker values were not normally distributed and contained outliers, we eliminated those influential observations that had Cook’s distance > 1 [51]. Correlation analysis revealed robust negative associations between premorbid openness and the levels of tau, *p*tau-181, tau/Aβ_1–42_ and *p*tau-181/Aβ_1–42_. Likewise, premorbid extraversion correlated negatively with tau, *p*tau-181, tau/Aβ_1–42_ and *p*tau-181/Aβ_1–42_ biomarker concentrations.

**Table 2:** Kendall’s correlations between premorbid personality domains and biomarkers

	Neuroticism	Extraversion	Openness	Agreeableness	Conscientiousness
Aβ_1–42_	0.08	0.03	0.07	–0.02	0.04
tau	0.03	–0.17**	–0.25***	–0.05	–0.06
*p*tau-181	0.01	–0.15**	–0.25***	0.00	–0.07
tau/Aβ_1–42_	0.00	–0.17**	–0.24***	–0.04	–0.01
*p*tau-181/Aβ_1–42_	–0.03	–0.16*	–0.25***	–0.01	–0.01

*p < 0.05, **p < 0.01, ***p < 0.001

*Aβ* amyloid beta, p*tau* phosphorylated tau

### Personality characteristics, CSF biomarkers and cognitive performance

Hierarchical multiple regressions revealed that, after controlling for age, gender, education level, APOEε4 expression and cognitive status, the low Aβ_1–42_ concentrations (*β* = –0.281, *p* < 0.01), high tau/Aβ_1–42_ levels (*β* = 0.237, *p* < 0.01) and high *p*tau-181/Aβ_1–42_ levels (*β* = 0.422, *p* < 0.001) contributed significantly in predicting the CDR SoB score. The concentrations of tau and *p*tau-181 did not reach any significance in model loading.

In the third step, where ratings of each personality domain were considered, only openness to experience contributed significantly in predicting the CDR SoB score (in regression model with tau and Aβ_1–42_ , *β* = –0.19, *p* < 0.05; with *p*tau and Aβ_1–42﻿_ ,﻿ *β* = –0.266, *p* < 0.01; with tau/Aβ_1–42_ , *β* = –0.193, *p* < 0.05; with *p*tau/Aβ_1–42_ , *β* = –0.252, *p* < 0.01). More precisely, after controlling for age, gender, education level, APOEε4 expression, cognitive status (Controls/CI) and biomarker concentrations, low premorbid openness to experience was associated with high CDR SoB score, and thus low cognitive performance.

**Fig. 1:**
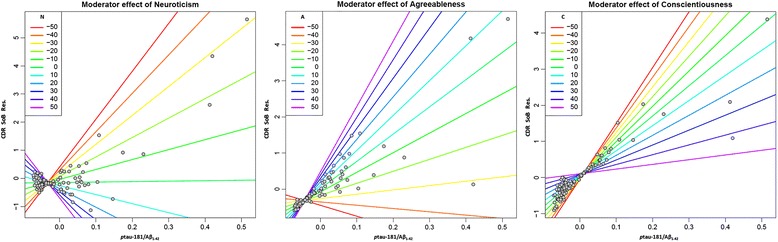
Significant premorbid personality and biomarker interaction effect on cognitive functioning. Dispersion diagram plot depicting the fit of a model with predicted cognitive performance score (*ordinate axis*) and an interaction between biomarker concentrations (*abscissa axis*) and premorbid personality. *Lines*, predicted centred values of premorbid personality traits. *Left*: with low neuroticism (*N*), greater *p*tau-181/Aβ_1–42_ concentrations predict lower CDR SoB scores, and thus better cognitive functioning. With relatively small *p*tau-181/Aβ_1–42_ value, the interaction effect is inversed. *Middle*: interaction between high agreeableness (*A*) level and high *p*tau-181/Aβ_1–42_ value predicts higher CDR SoB score, and thus lower cognitive functioning. Effect is inversed with relatively small *p*tau-181/Aβ_1–42_ concentrations. *Right:* at low conscientiousness (*C*) level, greater *p*tau-181/Aβ_1–42_ concentrations predict poorer cognitive performance (high CDR SoB score). With high C, smaller *p*tau-181/Aβ_1–42_ ratios predict better cognitive functioning (low CDR SoB score). *CDR SoB Res* residuals of clinical dementia rating Sum of Boxes score, *Aβ* amyloid beta, p*tau* phosphorylated tau

Furthermore, to investigate the modulation effect of premorbid personality on the cognitive performance–cerebral pathology relationship, we included the interactions between each CSF biomarker concentration and each personality domain in the last step of our model. The interactions between premorbid neuroticism and tau (*β* = –0.202, *p* < 0.05), premorbid neuroticism and tau/Aβ_1–42_ (*β* = –0.357, *p* < 0.01), and premorbid neuroticism and *p*tau-181/Aβ_1–42_ (*β* = –0.359, *p* < 0.01) concentrations negatively correlated with CDR SoB score. At high levels of neuroticism (i.e., 1 SD above mean) higher concentrations of tau, tau/Aβ_1–42_ and *p*tau-181/Aβ_1–42_ were related to low CDR SoB score. With low neuroticism (1 SD below mean), higher levels of the same biomarkers were associated with high CDR SoB score, and thus poor cognitive functioning (see Fig. 1).

Interactions between agreeableness and tau/Aβ_1–42_ concentrations (*β* = 0.299, *p* < 0.01) and between agreeableness and *p*tau-181/Aβ_1–42_ concentrations (*β* = 0.206, *p* < 0.01) contributed significantly to cognitive performance (Fig. 1). With high agreeableness, greater levels of those two biomarkers predicted a high score of CDR SoB, and thus poorer cognitive functioning; while with low agreeableness, higher biomarker concentrations predicted a lower CDR SoB score, and thus better cognitive functioning.

Finally, premorbid conscientiousness and Aβ_1–42_ (*β* = –0.182, *p* < 0.05) and conscientiousness and *p*tau-181/Aβ_1–42_ (*β* = –0.233, *p* < 0.05) interactions were shown to be significant in predicting CDR SoB. At high conscientiousness, lower CDR SoB score was predicted by higher *p*tau-181/Aβ_1–42_ levels and lower Aβ_1–42_ levels; while at low conscientiousness, greater *p*tau-181/Aβ_1–42_ levels and lower Aβ_1–42_ levels predicted a higher CDR SoB score, and thus worse cognitive functioning. Figure 1 graphically shows the relationship between neuroticism, agreeableness and conscientiousness and *p*tau-181/Aβ_1–42_ as well as CDR SoB score as examples of premorbid personality and biomarkers’ interaction effect on cognitive performance.

## Discussion

We have found that interactions between levels of premorbid neuroticism, conscientiousness and agreeableness and CSF biomarkers of AD pathology predict cognitive performance in healthy older participants and in subjects with MCI and mild AD dementia. Furthermore, lower premorbid openness independently predicted lower cognitive functioning after controlling for biomarker concentrations. To our knowledge, our study is the first to investigate the impact of premorbid personality on the relationship between cognitive functioning and CSF biomarkers of AD pathology.

There were associations between specific premorbid personality traits and tau, *p*tau-181, tau/Aβ_1–42_ and *p*tau-181/Aβ_1–42_ ratios, but we did not find any association with the Aβ_1–42_ level. While tau/Aβ_1–42_ and *p*tau-181/Aβ_1–42_ ratios may be considered markers of concomitant cerebral amyloid pathology and tau-related neurodegeneration, their associations with personality traits seem to be largely due to associations with the tau and *p*tau-181 levels. One possible explanation for these findings is that, in the clinical stages of AD, pathological changes of CSF Aβ_1–42_ may have reached the maximum level and remain relatively stable, while changes in tau and *p*tau-181 concentrations are still ongoing and more closely associated with cognitive performance [8]. Alternatively, premorbid personality characteristics may contribute to cognitive reserve rather than directly to the development of cerebral amyloid pathology. In this way, premorbid personality would modify the magnitude and dynamics of cognitive impairment related to neurodegeneration and tau pathology.

Furthermore, we observed that the effect of CSF biomarkers on cognitive performance change depending on the level of specific premorbid personality traits. More precisely, at low levels of neuroticism, higher concentrations of tau and higher tau/Aβ_1–42_ and *p*tau-181/Aβ_1–42_ ratios predicted poorer cognitive performance. At low premorbid conscientiousness, higher Aβ_1–42_ and lower *p*tau-181/Aβ_1–42_ ratios predicted worse cognitive functioning, suggesting a protective effect of conscientiousness. These findings are partly in line with a clinical and autopsy study [20], reporting premorbid conscientiousness to be significantly higher in dementia patients with cerebral pathology compared with non-demented individuals confirmed at autopsy to have had cerebral AD pathology. High neuroticism, however, acted as a vulnerability factor for AD [20]. In our study, higher levels of neuroticism predicted worse cognitive functioning only when the CSF tau levels and the tau/Aβ_1–42_ and *p*tau-181/Aβ_1–42_ ratios are relatively low (i.e. suggesting the absence of an AD biomarker profile). This might suggest that there is a “cutoff point” related to cerebral pathology after which the interaction effect on cognitive performance reverses. Finally, premorbid agreeableness moderated the biomarker–cognitive performance relationship so that the high tau/Aβ_1–42_ and *p*tau-181/Aβ_1–42_ concentrations predicted worse cognitive functioning when agreeableness was high. Different results might be due to different study designs applied. Moreover, our sample was comprised of controls and patients with MCI or mild AD dementia unlike in the clinical-autopsy study, where non-demented subjects were compared with participants with dementia. Noteworthily, premorbid neuroticism, conscientiousness and agreeableness considered independently did not predict cognitive functioning and the observed interaction effects on cognition were weak. This finding may be due to the fact that participants with cognitive impairment were at different stages of cognitive impairment and the age of clinical onset was not considered. Overall, the effects found in our sample need confirmation in further studies.

Our study also reveals a substantial premorbid personality role in distinguishing between the CI and control groups because the two groups differed significantly in the domain of openness. This finding may seem less expected because clinical AD symptoms, such as troublesome behaviour [53] and anxiety [54], and dementia risk [17, 18, 20, 55, 56] have been described as mainly related to high neuroticism and low conscientiousness. Only a few studies estimate the openness to experience—a trait which refers to cognitive curiosity and activity, imagination, sensitivity to culture and arts, and behavioural flexibility [57]—to play an important role in predicting dementia or AD. Duberstein et al. [58] found that AD dementia risk was greater among subjects who had not only higher neuroticism and lower conscientiousness, but also lower openness to experience. Furthermore, a longitudinal study comparing personality traits in normal ageing and MCI showed that MCI subjects had lower premorbid openness compared with healthy controls [59].

Moreover, our results revealed that premorbid openness predicts cognitive performance beyond the subject’s cognitive status, demographic variables, APOEε4 status and CSF biomarker level. In our study, openness was the only personality domain from the FFM which contributed independently to cognitive performance. This finding is in line with results from some previous studies. For instance, Terry et al. [60] showed that openness, and not neuroticism, was associated with better memory performance in older adults with questionable dementia, even after controlling for socio-demographic variables and cognitive functioning [60]. However, no premorbid personality assessment was performed and possible personality changes related to dementia were not considered in this study. Furthermore, Chapman et al. [61] revealed that in older subjects not only higher extraversion and neuroticism but also lower openness predicted worse average cognitive function over 7 years. Persons who are more open dispose a greater intellectual curiosity and have lifelong patterns of cognitive activity, which probably facilitates processing of new information, helps maintain cognitive functions [62] and leads to better cognitive reserve [63]. Sharp et al. [64] explain the implication of openness in sustained cognitive functioning in terms of preserved differentiation theory. This view suggests that people who were more mentally active throughout their lives show superior preservation of their baseline cognitive activity while, according to the differential preservation hypothesis, greater mental activity not only enhances the level of cognitive performance but also slows the trajectory of age-related cognitive decline [65]. In a longitudinal study, subjects with higher levels of openness in the second half of their life span had higher performance across all cognitive tests and scored less for cognitive decline, even after controlling for education and activities of daily living, but did not alter the trajectories of cognitive performance over age, supporting the preserved differentiation theory [64]. Along with these previous findings, our results strongly suggest an implication of premorbid openness in predicting cognitive functioning in older persons. Conforming with our results, higher premorbid openness to experience might be one of the patterns of a resilient personality profile which boost subjects’ cognitive reserve and may act as a protecting factor against cognitive decline.

Inheritance of the APOEε4 allele is considered a major genetic risk factor for non-familial AD [66–68]. As for genotype–personality phenotype interactions, neuroticism and extraversion were recently found to act as moderators on the associations between the APOEε4 status and the two outcomes; that is, worse cognitive function and incidence of AD dementia over a span of 6.5 years [28]. More precisely, the carriers of APOEε4 with high scores on neuroticism and extraversion had poorer cognitive functioning and higher incidence of AD compared with APOEε4 carriers with lower neuroticism and extraversion scores. Biomarkers of cerebral pathology were not included in this study, however. In our study, APOEε4 status differed across clinical and control groups, but was not a significant predictor for cognitive functioning.

The main strengths of our study are the use of validated instruments, the comprehensive personality and clinical assessment, and the inclusion of factors with established effects on the relationship between cerebral pathology and clinical manifestations. Neuroticism, conscientiousness and extraversion—the domains widely described in the literature to differ between cognitively impaired subjects and healthy controls—did not predict cognitive performance alone. The absence of associations may be due to a small sample size and thus having a reduced statistical power compared with results obtained from larger cohorts. Furthermore, the assessment of personality characteristics was made by the subjects’ proxies and might have been biased by their relationship with the participants. It should be noted that there is a good agreement between self-report and informant-report ratings [69], but informant ratings tend to discriminate more sensitively healthy control and demented groups compared with self ratings [55]. In addition, evaluating a subject’s premorbid personality retrospectively may be less precise than prospective personality evaluation due to potential memory bias, and may be influenced by the impact of the cognitive decline on the personal relationship between caregiver and patient. The preclinical stage of AD is characterized by developing AD pathology in the absence of symptoms. During the transition phase to clinical AD, there is a gradual decline in cognition that can be paralleled by subtle changes in non-cognitive clinical features. The continuous progression from the preclinical to the prodromal stage makes it impossible to define a precise onset of the clinical state [70]. There is very little evidence on the timeline of personality change in relation to the onset of cognitive impairment, especially considering biomarkers of AD pathology. While several studies have shown that subjective cognitive complaints predict cognitive decline and are associated with AD pathology, it is not clear how early personality changes start. A recent study found that subjects with MCI and high cerebral Aβ burden expressed more burdensome coping strategies, dismissive attitudes and dependency comparative with healthy controls. In the same study, cognitively normal participants with high Aβ burden had more subjective cognitive complains than cognitively normal participants with low/normal Aβ burden, but did not express different coping strategies, dismissive attitudes and dependency [71]. In this context, we consider it unlikely that substantial prodromal personality changes occur more than 5 years before the onset of cognitive decline. However, we cannot exclude the possibility that subtle personality changes occur even earlier.

## Conclusions

The interaction between premorbid neuroticism, conscientiousness and agreeableness and cerebral pathology as measured by CSF biomarkers predicts cognitive functioning. Subjects with MCI and mild dementia have lower premorbid openness compared with healthy controls. Premorbid openness predicts cognitive impairment independently of cerebral AD pathology. Further studies with larger samples are needed in order to confirm these findings and their potential implications for prevention of cognitive decline, and to better understand the nature of premorbid personality in relation to brain pathology and cognitive performance.

## Appendix 1

**Table 3:** Description of NEO-PI-R personality domains and facets scales

Neuroticism: identifies individuals who are prone to psychological distress
N1. Anxiety: level of free floating anxiety
N2. Angry Hostility: tendency to experience anger and related states such as frustration and bitterness
N3. Depression: tendency to experience feelings of guilt, sadness, despondency and loneliness
N4. Self-Consciousness: shyness or social anxiety
N5. Impulsiveness: tendency to act on cravings and urges rather than reining them in and delaying gratification
N6. Vulnerability: general susceptibility to stress
Extraversion: quantity and intensity of energy directed outwards into the social world
E1. Warmth: interest in and friendliness towards others
E2. Gregariousness: preference for the company of others
E3. Assertiveness: social ascendancy and forcefulness of expression
E4. Activity: pace of living
E5. Excitement Seeking: need for environmental stimulation
E6. Positive Emotions: tendency to experience positive emotions
Openness to Experience: the active seeking and appreciation of experiences for their own sake
O1. Fantasy: receptivity to the inner world of imagination
O2. Aesthetics: appreciation of art and beauty
O3. Feelings: openness to inner feelings and emotions
O4. Actions: openness to new experiences on a practical level
O5. Ideas: intellectual curiosity
O6. Values: readiness to re-examine own values and those of authority figures
Agreeableness: the kinds of interactions an individual prefers from compassion to tough mindedness
A1. Trust: belief in the sincerity and good intentions of others
A2. Straightforwardness: frankness in expression
A3. Altruism: active concern for the welfare of others
A4. Compliance: response to interpersonal conflict
A5. Modesty: tendency to play down own achievements and be humble.
A6. Tender-Mindedness: attitude of sympathy for others.
Conscientiousness: degree of organization, persistence, control and motivation in goal directed behavior
C1. Competence: belief in own self efficacy
C2. Order: personal organization
C3. Dutifulness: emphasis placed on importance of fulfilling moral obligations
C4. Achievement Striving: need for personal achievement and sense of direction
C5.Self-Discipline: capacity to begin tasks and follow through to completion despite boredom or distractions.
C6. Deliberation: tendency to think things through before acting or speaking.

Exerted and adapted from P.T. Costa, R.R. McCrae. Hogrefe Ltd. The Test People, Oxford. http://www.unifr.ch/ztd/HTS/inftest/WEBInformationssystem/en/4en001/d590668ef5a34f17908121d3edf2d1dc/hb.htm

## Abbreviations

AD: Alzheimer’s disease
ADL: Activities of daily living
APOEε4: ε4 allele of Apolipoprotein E
Aβ: Amyloid beta
CDR: Clinical Dementia Rating Scale
CI: Cognitively impaired
CSF: Cerebrospinal fluid
DSM: Diagnostic and Statistical Manual of Mental Disorders
FFM: Five-Factor Model
HAD: Hospital Anxiety and Depression Scale
IADL: Instrumental activities of daily living
IQCODE: Informant Questionnaire on Cognitive Decline in the Elderly
MMSE: Mini-Mental State Examination
NEO-PI-R: Revised NEO Personality Inventory, Form R
ptau: Phosphorylated tau
SD: Standard deviation
tau: total tau
